# Clinical efficacy and safety of combination therapy of tocilizumab and steroid pulse therapy for critical COVID-19 in HD patients

**DOI:** 10.1007/s10157-021-02126-4

**Published:** 2021-08-26

**Authors:** Masataro Toda, Kentaro Fujii, Ayumi Yoshifuji, Yasushi Kondo, Kazuto Itoh, Kazuhiko Sekine, Takahide Kikuchi, Munekazu Ryuzaki

**Affiliations:** 1 Department of Nephrology, Tokyo Saiseikai Central Hospital, 1-4-17 Mita, Minato-ku, Tokyo, 108-0073 Japan; 2 Department of Rheumatology, Keio University School of Medicine, Tokyo, Japan; 3 Department of General Internal Medicine, Tokyo Saiseikai Central Hospital, Tokyo, Japan; 4 Department of Emergency and Critical Care Medicine, Tokyo Saiseikai Central Hospital, Tokyo, Japan; 5 Department of Hematology, Tokyo Saiseikai Central Hospital, Tokyo, Japan

**Keywords:** Tocilizumab, Steroid pulse therapy, COVID-19, Hemodialysis, Cytokine storms

## Abstract

**Background:**

Critical coronavirus disease 2019 (COVID-19) has a high fatality rate, especially in hemodialysis (HD) patients, with this poor prognosis being caused by systemic hyperinflammation; cytokine storms. Steroid pulse therapy or tocilizumab (TCZ) have insufficient inhibitory effects against cytokine storms in critical cases. This study evaluated the clinical effects and safety of combining steroid pulse therapy and TCZ.

**Methods:**

From September 2020 to May 2021, 201 patients with COVID-19 were admitted to our hospital. Before February 2021, patients with an oxygen demand exceeding 8 L/min were intubated and treated with standard therapy (dexamethasone and antiviral therapy). After February 2021, patients underwent high-flow nasal cannula oxygen therapy and were treated with TCZ (8 mg/kg) and methylprednisolone (mPSL) (500 mg/day [≤ 75 kg], 1000 mg/day [> 75 kg]) for 3 days. We compared background characteristics, laboratory findings, and prognosis between non-HD and HD patients and between patients who received and did not receive TCZ and mPSL pulse therapy.

**Results:**

Among non-HD patients, the TCZ + mPSL pulse group had significantly higher survival rates and lower secondary infection rates (*p* < 0.05), than the standard therapy group. All HD patients in the standard therapy group with oxygen demand exceeding 8 L/min died. Contrastingly, all patients in the TCZ + mPSL pulse group survived, with their oxygen demand decreasing to 0–1 L/min within 3 weeks post-administration.

**Conclusion:**

TCZ combined with mPSL pulse therapy improved the survival rate without significant adverse events in critical HD and non-HD patients with COVID-19 by strongly suppressing systemic hyperinflammation.

## Introduction

In December 2019, the first case of coronavirus disease 2019 (COVID-19), which is caused by the novel severe acute respiratory syndrome coronavirus 2, was reported in Wuhan, China; subsequently, it has dramatically spread worldwide. Although most patients with COVID-19 present with mild symptoms, 14% and 5% of the patients demonstrate respiratory failure and require critical care, respectively. Further, the fatality rate of critical patients with COVID-19 reaches up to 40% [1]. There are numerous active studies on effective treatments for COVID-19, with dexamethasone [2] and remdesivir [3] being currently recommended as standard therapy based on COVID-19 treatment guidelines published by the National Institutes of Health.

Despite the recent progress in COVID-19 treatment, the COVID-19 prognosis in hemodialysis (HD) patients remains poor. Previous studies have shown that patients with kidney disease have worse COVID-19 outcomes [4], especially in HD patients with the mortality rate of COVID-19 in HD patients being 14% [5] and reaching 50% among critical cases [6]. This could be generally attributed to HD patients being more susceptible to severe infections given their compromised immune system caused by damage to neutrophils, lymphocytes, and macrophages. Additionally, comorbid underlying diseases, including diabetes mellitus, in HD patients contribute to a further decline in immunity [7]. Therefore, there is a need to establish effective therapeutic strategies against critical COVID-19 in HD patients.

In patients with severe or critical COVID-19, clinical symptoms rapidly worsen due to cytokine storms, which involve systemic hyperinflammation caused by uncontrolled cytokine overproduction and causes multiple organ failure [8]. Immunomodulators, including steroids, and various cytokine receptor inhibitors have been used to suppress cytokine storms in patients with COVID-19. The RECOVERY trial, which demonstrated strong efficacy of dexamethasone, encouraged reevaluation of the efficacy of systemic steroid administration; subsequently, dexamethasone has become the standard treatment [2]. Furthermore, there have been several randomized clinical trials (RCTs) on the efficacy of steroid pulse therapy with the expectation of a higher immunosuppressive effect, as well as the utility of steroid pulse therapy [9–11]. IL-6 are crucially involved in the cytokine storm; further, recent studies have indicated the efficacy of tocilizumab (TCZ), which is an IL-6 receptor inhibitor [12]. It is difficult to suppress cytokine storms in critical patients with COVID-19, especially HD patients, which causes poor outcomes at our hospital. Accordingly, we began treating patients using combination therapy of tocilizumab and steroid pulse therapy, with an expectation of strong suppression of the initial phase of the cytokine storm. This study aimed to evaluate the clinical efficacy and safety of combination therapy involving TCZ and steroid pulse therapy in HD and non-HD patients.

## Materials and methods

A total of 201 patients with COVID-19 were admitted to our hospital from September 2020 and May 2021. All patients with severe or critical COVID-19 were treated using antiviral therapy (glomerular filtration rate [GFR] ≥ 30, remdesivir; GFR < 30, favipiravir) and dexamethasone as standard therapy. From September 2020 to January 2021, patients whose oxygen demand with oxygen mask (the oxygen dose was increased and decreased by 1 L/min when SpO_2_ was < 94% and > 97%, respectively) exceeded 8 L/min were intubated, placed on a ventilator, and treated using standard therapy. After February 2021, patients whose oxygen demand exceeded 8 L/min underwent high-flow nasal cannula (HFNC) oxygen therapy and were treated using 8 mg/kg TCZ and methylprednisolone (mPSL) (500 mg/day and 1000 mg/day for < 75 kg and > 75 kg in body weight, respectively) for 3 days, followed by administration of an adequate dexamethasone dose. In case this was insufficient, the patients were intubated. Moreover, if the aforementioned conditions for intubation were met but there was no consent for intubation, the patient only underwent HFNC. Among patients requiring > 8 L/min of oxygen, there were 17 and 5 non-HD and HD patients, respectively. Each group was divided into those who received standard therapy (standard therapy group) and those who received combination therapy of TCZ and steroid pulse therapy (TCZ + mPSL pulse group). Further, we performed between-group comparisons of the background characteristics, comorbidities, laboratory findings on admission day, and prognosis. Median values were compared using the Mann–Whitney *U* test. Statistical significance was set at *p* < 0.05.

## Results

### Combination therapy of tocilizumab and steroid pulse therapy was safe and effective for critical non-HD patients with COVID-19

Among 190 non-HD patients with COVID-19 admitted to our hospital from September 2020 to May 2021, 17 cases showed an oxygen demand exceeding 8 L/min. Further, eight and nine patients were treated using combination therapy (TCZ and steroid pulse therapy) and standard therapy, respectively. In both groups, patients received dexamethasone starting at 6 mg, with the dose being gradually increased or decreased as required. All patients in the TCZ + mPSL pulse group received remdesivir; further, three and six patients in the standard therapy group received favipiravir and remdesivir, respectively. All patients in both groups were intubated or placed on HFNC when the oxygen demand exceeded 8 L/min. In the standard therapy group, four patients died (29.25 days from admission on average) while five patients survived and were discharged or transferred (34.4 days from admission on average). On the other hand, all patients in the TCZ + mPSL pulse group survived and were discharged (21.1 days from admission on average) (Table 1).

**Table 1:** Prognosis of critical COVID-19 patients whose oxygen demand exceeded 8 L

	No.	Age sex	Onset to O_2_ 8 L (days)	COVID-19 severity	Treatment	Respiratory management or peak O_2_ demand (L)	Outcome
Standard care	1	93 F	7	Critical	FPV 1600 mg (14 days), Dex	Intubate	Death (day 45)
	2	86 M	3	Critical	FPV 1600 mg (14 days), Dex	Intubate	Death (day 30)
	3	76 M	8	Critical	RDV 100 mg (until death), Dex	Intubate	Death (day 21)
	4	64 M	10	Critical	RDV 100 mg (6 days*), Dex	Intubate (ECMO)	Death (day 21)
	5	72 F	12	Critical	RDV 100 mg (10 days), Dex	Intubate	Discharge (day 30)
	6	77 M	7	Critical	RDV 100 mg (10 days), Dex	Intubate	Transfer (day 37)
	7	82 M	8	Critical	FPV 1600 mg (9 days), Dex	HFNC	Transfer (day 46)
	8	86 F	13	Critical	RDV 100 mg (10 days), Dex	HFNC	Transfer (day 35)
	9	58 M	10	Critical	RDV 100 mg (10 days), Dex	HFNC	Discharge (day 24)
Tocilizumab mPSL pulse	1	54 M	9	Critical	RDV 100 mg (10 days), Dex tocilizumab, mPSL 1 g 3 days	HFNC	Discharge (day 28)
	2	70 M	4	Critical	RDV 100 mg (7 days), Dex tocilizumab, mPSL 1 g 3 days	Intubate	Discharge (day 33)
	3	83 M	5	Critical	RDV 100 mg (10 days), Dex tocilizumab, mPSL 0.5 g 3 days	HFNC	Transfer (day 21)
	4	58 M	8	Critical	RDV 100 mg (10 days), Dex tocilizumab, mPSL 1 g 3 days	HFNC	Discharge (day 24)
	5	56 M	15	Critical	RDV 100 mg (5 days), Dex tocilizumab, mPSL 1 g 3 days	HFNC	Discharge (day 18)
	6	61 M	4	Crtitical	RDV 100 mg (10 days), Dex tocilizumab, mPSL 1 g 3 days	HFNC	Discharge (day 18)
	7	86 M	15	Critical	RDV 100 mg (10 days), Dex tocilizumab, mPSL 0.5 g 3 days	HFNC	Transfer (day 14)
	8	43 M	9	Critical	RDV 100 mg (10 days), Dex tocilizumab, mPSL 1 g 3 days	HFNC	Discharge (day 14)

*RDV* remdesivir, *FPV* favipiravir, *Dex* dexamethasone, *mPSL* methylprednisolone, *HFNC* high-flow nasal cannula, *ECMO* extracorporeal membrane oxygenation 6 days

*Discontinued on day 6 because of renal injury

The survival rate and secondary infection rate were significantly higher and lower, respectively, in the TCZ + mPSL pulse group than in the standard therapy group (*p* < 0.05). There were no significant between-group differences in background characteristics, comorbidities, duration from onset to hospitalization or oxygen demand > 8 L/min, and laboratory data at admission (Table 2).

**Table 2:** Background, comorbidity and laboratory data of critical COVID-19 patients

		Standard care *N* = 9	TCZ + mPSL pulse *N* = 8	*p* value
Background	Male, *n* (%)	6 (67)	8 (100)	0.072
	Median age (IQR)	77.0 (14.0)	59.5 (17.8)	0.062
	Median BMI (IQR)	24.0 (4.6)	28.4 (9.6)	0.491
	Median days from onset to admission (IQR)	7.0 (3.0)	7.5 (6.8)	0.906
	Median days to O_2_ 8 L (IQR)	8.0 (3.0)	8.5 (5.8)	0.944
Comorbidities *n* (%)	Any	8 (89)	7 (88)	0.929
	Diabetes	2 (22)	3 (38)	0.490
	Hypertention	4 (44)	6 (75)	0.201
	Hyperlipidemia	2 (22)	1 (13)	0.600
	Malignancy	1 (11)	2 (25)	0.453
	Cardiovascular disease	3 (33)	0 (0)	0.072
	Lung disease	1 (11)	1 (13)	0.929
	CKD	2 (22)	3 (38)	0.165
	Bronchial asthma	0 (0)	1 (13)	0.274
Lab data on admission day median (IQR)	WBC (/μL)	8200 (4100)	5650 (3900)	0.309
	Lymphocyte (/μL)	673.2 (556.8)	804.9 (598.3)	0.888
	Hb (g/dL)	13.4 (1.3)	14.3 (1.6)	0.556
	Plt (× 10^3^/μL)	212 (42)	216 (162)	0.888
	Cr (mg/dL)	0.93 (0.86)	1.12 (0.23)	0.724
	LDH (U/L)	590 (216)	475 (220)	0.423
	CK (U/L)	127 (66)	140 (302)	0.623
	CRP (mg/dL)	15.14 (3.79)	11.18 (13.08)	0.609
	Ferritin (ng/mL)	813.4 (801.0)	624.7 (588.6)	0.639
	PT (second)	13.2 (0.7)	13.2 (1.9)	0.760
	D-dimer (μg/mL)	1.7 (1.8)	1.7 (2.6)	0.939
	KL-6 (U/mL)	210 (51)	364 (320)	0.073
	Procalcitonin (ng/mL)	0.33 (0.38)	0.12 (0.14)	0.053
	BNP (pg/mL)	31.3 (57.8)	37.6 (46.9)	0.486
Outcome *n* (%)	Survive	5 (56)	8 (100)	0.031
	Infectious complication	4 (44)	0 (0)	0.031
	Bacteria	4 (44)	0 (0)	0.031
	Non-bacteria	1 (11)	0 (0)	0.331

*TCZ* tocilizumab, *mPSL* methylprednisolone, *BMI* body mass index, *CKD* chronic kidney disease

**Fig. 1:**
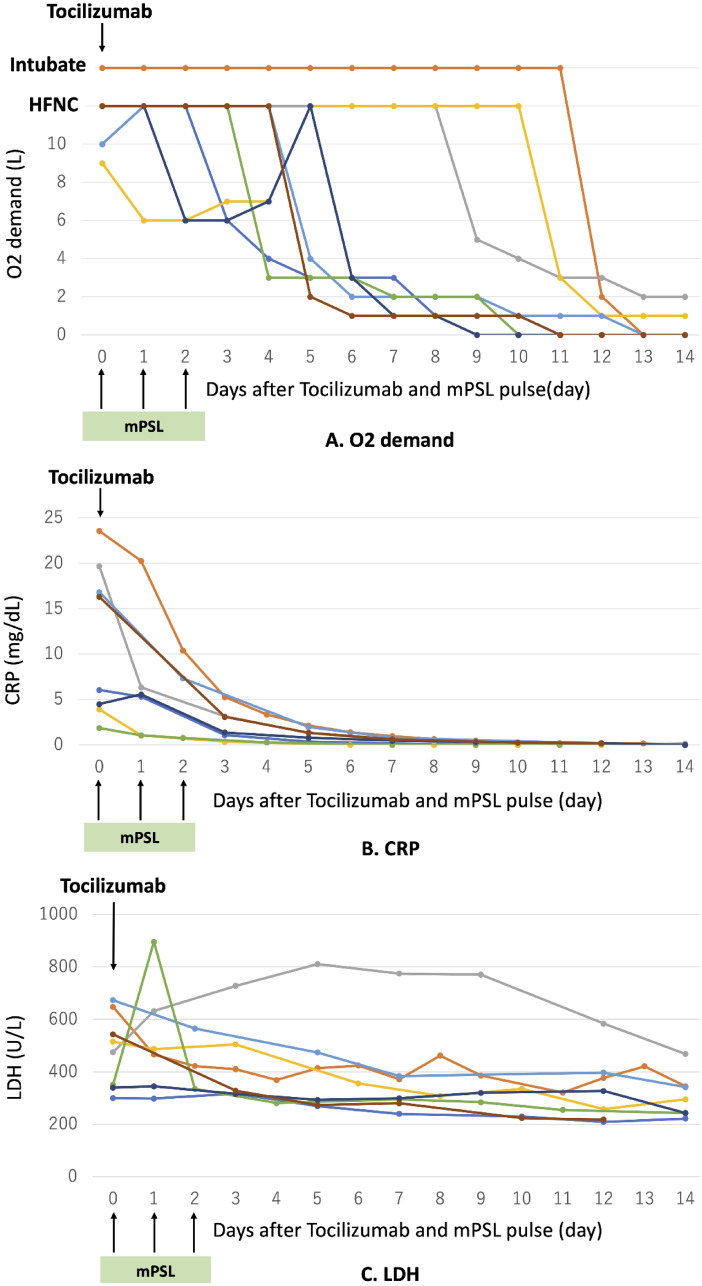
Clinical course of critical Covid 19 patient with TCZ + mPSL pulse. In all patients, CRP decreased markedly immediately after TCZ and mPSL pulse administration and was almost zero in all patients within 2 weeks. Along with the decrease in CRP, oxygen demand also decreased markedly in all patients and was only 0–2 L/min within 2 weeks. LDH also showed a decreasing trend. *TCZ* tocilizumab, *mPSL* methylprednisolone, *HFNC* high-flow nasal cannula, *CRP* c-reactive protein, *LDH* lactate dehydrogenase

All patients in the TCZ + mPSL pulse group showed a marked decrease in C-reactive protein (CRP) levels immediately after treatment, which reached almost zero in all patients within 2 weeks. Also, all patients showed a marked decrease in oxygen demand, which reached 0–2 L/min (0 L/min: 6 patients, 1 L/min: 1 patient, 2 L/min: 1 patient) within 2 weeks. Furthermore, lactate dehydrogenase (LDH) levels showed a decreasing trend (Fig. 1).

### Combination therapy of tocilizumab and steroid pulse therapy was effective for critical HD patients with COVID-19

We admitted 11 HD patients to our hospital between September 2020 and May 2021. Among them, nine and two patients were treated using standard therapy and combination therapy (TCZ and mPSL pulse therapy). In the standard therapy group, three patients whose oxygen demand exceeded 8 L/min required respiratory management (two underwent HFNC therapy and one was intubated) while six patients were treated using oxygen masks alone. In the TCZ + mPSL pulse group, one patient required respiratory management and was placed on HFNC. All patients in both groups received dexamethasone as standard therapy starting at 6 mg, which was gradually increased or decreased as required, as well as favipiravir as the antiviral therapy. In the standard therapy group, all patients who were treated using oxygen masks alone survived and were discharged (23.7 days from admission on average). Further, all patients who received respiratory management in the standard therapy group died (33.3 days from admission on average). Contrastingly, all patients in the TCZ + mPSL pulse group survived and were discharged or transferred (40.0 days from admission on average) (Table 3). Among patients who received respiratory management, there was no between-group difference in background characteristics, comorbidities, or days from onset to oxygen demand exceeding 8 L/min; further, laboratory findings at admission were slightly worse in the standard therapy group while the duration from onset to hospitalization was longer in the TCZ + mPSL pulse group (Table 4).

**Table 3:** Prognosis of critical COVID-19 patients undergoing HD

		No.	Age sex	Causes of CKD	COVID-19 Severity	Treatment	Respiratory Management or Peak O_2_ demand (L)	Outcome
Standard care	Oxygen mask	1	49 M	Sclerosis	Severe	FPV 1600 mg (7 days), Dex	6	Discharge (day 25)
		2	58 M	Sclerosis	Severe	FPV 1600 mg (7 days), Dex	5	Discharge (day 23)
		3	57 M	Sclerosis	Severe	FPV 1600 mg (14 days), Dex	4	Discharge (day 23)
		4	80 F	Sclerosis	Severe	FPV 1600 mg (7 days), Dex	2	Transfer (day 39)
		5	56 F	Sclerosis	Severe	FPV 1600 mg (7 days), Dex	1	Discharge (day 21)
		6	55 M	MN	Severe	FPV 1600 mg (7 days), Dex	1	Discharge (day 11)
	Respiratory management	1	72 M	DM	Critical	FPV 1600 mg (14 days), Dex	HFNC	Death (day 48)
		2	70 M	DM	Critical	FPV 1600 mg (14 days), Dex	Intubate	Death (day 18)
		3	86 F	DM	Critical	FPV 1600 mg (14 days), Dex tocilizumab	HFNC	Death (day 34)
Tocilizumab mPSL pulse		1	73 F	DM	Critical	FPV 1600 mg (14 days), Dex tocilizumab, mPSL 0.5 g 3 days	HFNC	Transfer (day 54)
		2	73 M	DM	Critical	FPV 1600 mg (14 days), Dex tocilizumab, mPSL 0.5 g 3 days	8	Discharge (day 26)

*HD* hemodialysis, *CKD* chronic kidney disease, *MN* membranous nephropathy, *DM* diabetes mellitus, *FPV* favipiravir, *Dex* dexamethasone, *mPSL* methylprednisolone, *HFNC* high-flow nasal cannula

**Table 4:** Background, comorbidity and laboratory data of critical COVID-19 patients undergoing HD

		Standard care		TCZ + mPSL pulse (*n* = 2)
		Oxygen mask (*n* = 6)	Respiratory management (*n* = 3)	
Background	Male, *n* (%)	4 (67)	2 (67)	1 (50)
	Median age (IQR)	56.5 (2.5)	72.0 (8.0)	73.0 (0.0)
	Median BMI (IQR)	22.6 (11.1)	23.2 (1.1)	20.1 (1.4)
	Median days from onset to admission (IQR)	3.0 (2.3)	3.0 (1.0)	1.0 (0.0)
	Median days to O_2_ 8 L (IQR)		8.0 (6.0)	10.5 (2.5)
Comorbidities *n* (%)	Any	6 (100)	3 (100)	2 (100)
	Diabetes	0 (0)	3 (100)	2 (100)
	Hypertention	6 (100)	1 (33)	2 (100)
	Hyperlipidemia	2 (33)	0 (0)	1 (50)
	Malignancy	1 (17)	1 (33)	1 (50)
	Cardiovascular disease	4 (67)	2 (67)	1 (50)
	Lung disease	0 (0)	0 (0)	0 (0)
	CKD	6 (100)	3 (100)	2 (100)
	Bronchial asthma	0 (0)	0 (0)	0 (0)
Lab data on admission day median (IQR)	WBC (/μL)	3100 (525)	5300 (1250)	4850 (50)
	Lymphocyte (/μL)	662.8 (519.3)	468.0 (262.3)	1419.1 (174.5)
	Hb (g/dL)	10.3 (0.6)	11.6 (1.4)	11.4 (0.1)
	Plt (× 10^3^/μL)	131 (16)	106 (23)	151 (9.5)
	Cr (mg/dL)	9.75 (2.17)	11.75 (2.43)	8.63 (3.47)
	LDH (U/L)	346 (146)	358 (148)	187 (23)
	CK (U/L)	102 (57)	551 (421)	95 (15)
	CRP (mg/dL)	9.71 (15.92)	13.53 (10.01)	0.28 (0.12)
	Ferritin (ng/mL)	359.2 (328.1)	176.2 (905)	229.3 (191.4)
	PT (second)	12.0 (3.6)	13.4 (1.2)	13.1 (0.6)
	D-dimer (μg/mL)	1.6 (1.0)	1.7 (3.8)	1.7 (0.4)
	KL-6 (U/mL)	301 (65)	246 (81)	380 (18)
	Procalcitonin (ng/mL)	1.49 (3.33)	2.18 (2.05)	0.23 (0.03)
	BNP (pg/mL)	156.6 (136.8)	179.0 (430.4)	204.0 (160.8)
Outcome *n* (%)	Survive	6 (100)	0 (0)	2 (100)
	Infectious complication	1 (17)	2 (67)	1 (50)
	Bacteria	1 (17)	2 (67)	1 (50)
	Non-bacteria	0 (0)	1 (33)	0 (0)

*TCZ* tocilizumab, *mPSL* methylprednisolone, *BMI* body mass index, *CKD* chronic kidney disease

### The clinical course of critical HD patients with COVID-19 in the TCZ + mPSL pulse group

**Fig. 2:**
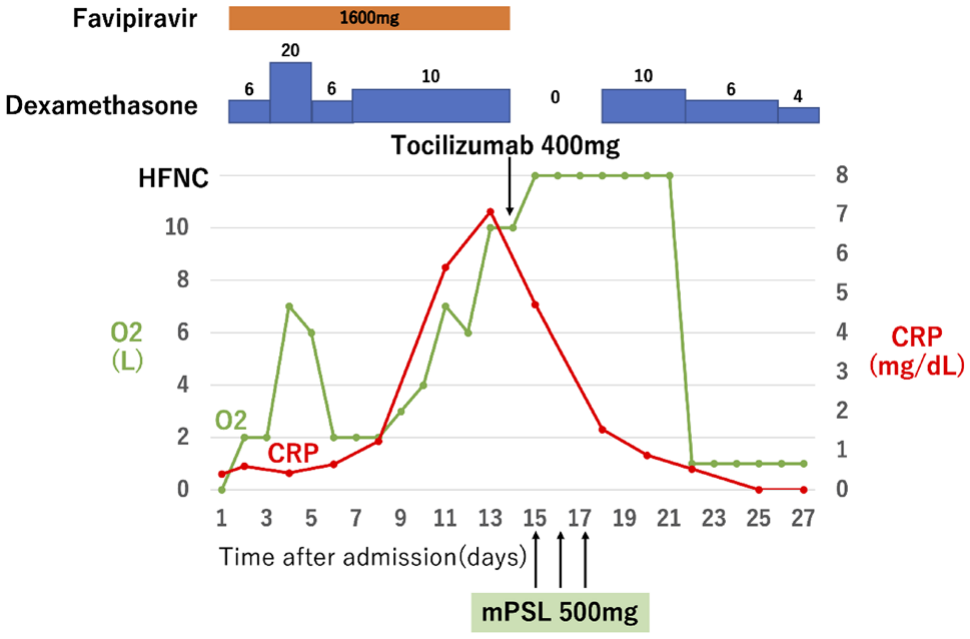
Clinical course of patient 1. Patients’ oxygen demand increased from zero when admitted to the hospital as their CRP increased, so placed on HFNC on day 14 of admission. Tocilizumab was administered on day 14 and mPSL pulse (500 mg/day) was performed on days 15–17. Immediately after tocilizumab and mPSL pulse administration, CRP decreased markedly, accompanied by a marked decrease in oxygen demand to 1 L/min with oxygen mask. *mPSL* methylprednisolone, *HFNC* high-flow nasal cannula, *CRP* c-reactive protein

**Fig. 3:**
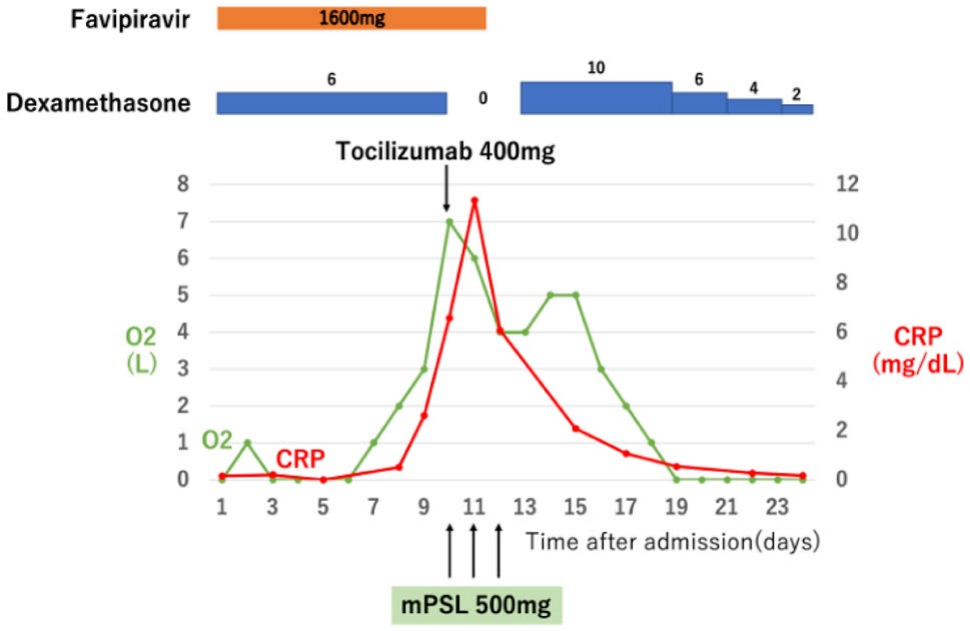
Clinical course of patient 2. Patients’ oxygen demand increased from zero when admitted to the hospital to 8 L/min as their CRP increased. Tocilizumab was administered on day 10 and mPSL pulse (500 mg/day) was performed on days 10–12. Immediately after tocilizumab and mPSL pulse administration, CRP decreased markedly, accompanied by a marked decrease in oxygen demand to 0 L/min on day 19. *mPSL* methylprednisolone, *HFNC* high-flow nasal cannula, *CRP* c-reactive protein

In this group, two patients did not require oxygen demand upon admission. Among them, one patient showed an oxygen demand exceeding 8 L/min on day 13 from admission. This patient was placed on HFNC on day 14, with TCZ and mPSL pulse (500 mg/day) being administered on day 14 and days 15–17, respectively. Subsequently, there was a marked decrease in CRP levels, with an accompanying marked decrease in oxygen demand. Further, on day 22, the patient was weaned off the HFNC and placed under an oxygen mask with an oxygen demand of 1 L/min (Fig. 2). For the second patient, the oxygen demand exceeded 8 L/min on day 10. The patient received TCZ and mPSL pulse (500 mg/day) on day 10 and days 10–12, respectively. Subsequently, there was a marked decrease in CRP levels, with an accompanying marked decrease in the oxygen demand to 0 L/min on day 19 (Fig. 3). Both patients were discharged or transferred and showed favorable outcomes.

## Discussion

Our findings showed that combination therapy of TCZ and steroid pulse therapy for critical HD and non-HD patients with COVID-19 resulted in a good prognosis without significant adverse events. Among non-HD patients, there was a significantly higher survival rate in the TCZ + mPSL pulse group than in the standard therapy group. Regarding the pathophysiology of critical COVID-19, there is excessive activation of acquired immunity, which results in uncontrolled excessive production of inflammatory cytokines, cytokine storm [13]. This leads to vascular endothelial damage and coagulation abnormalities, which causes multiple organ failure [14]. COVID-19 symptoms rapidly become critical 7–10 days after onset [15], which is associated with an increase in acute-phase response markers, including CRP and ferritin, and inflammatory cytokines, including IL-6, IL-2, and TNF-α [14, 16]. Contrastingly, there was a decrease in the viral load during this period [15], suggesting severe clinical symptoms in patients with severe-to-critical COVID-19 are caused by cytokine storms; therefore, there is a need for appropriate suppression of the cytokine storm [13].

Our critical cases showed exacerbated oxygen demand, with a marked increase in the inflammatory response after 9.78 days and 8.65 days from onset in HD and non-HD patients, respectively, which was consistent with previous reports [15]. Steroids suppress cytokine storms by inhibiting multiple inflammatory targets; exerting immunosuppressive effects on immune cells; and suppressing the production of key inflammatory molecules, including prostaglandins and leukotrienes [8]. Recently, McGroder et al. reported that 72% of patients with lung fibrosis required a ventilator. Fibrosis-related factors include the severity of the initial lung lesions and the ventilator duration [17]; therefore, it is crucial to strongly inhibit the initial inflammatory response and to prevent early-phase lung fibrosis. Although numerous RCTs have demonstrated the efficacy of steroids, the steroid type and dose remain unclear [18]. Regarding steroid pulse therapy, which strongly suppresses the inflammatory response, several studies have demonstrated the efficacy of moderate doses (mPSL 250 mg, 3 days) [9, 10]. Further, Tamura et al. suggested the treatment benefits of high doses (mPSL 1 g, 3 days) [11]. However, prolonged steroid use generally leads to susceptibility to infection and could cause severe or unusual infections due to immunosuppression [19]. Additionally, prolonged steroid use increases the risk of secondary infections, including bacterial and fungal infections, during hospitalization in COVID-19 patients [20]. Therefore, it is important to shorten the steroid treatment duration.

In COVID-19, cytokine storms are triggered by the action of IFNγ, TNFα, IL-1, IL-2, and IL-6. Non-survivors present with higher IL-6 levels than survivors [21]; further, IL-6 levels increase with illness deterioration [22]. Moreover, higher IL-6 levels are associated with a higher intubation risk [23]. IL-6 is crucially involved in the cytokine storm by promoting the production of various acute-phase proteins in hepatocytes as well as inducing B and T cell differentiation [24]. Gordon et al. reported that TCZ significantly improved the survival rate and shortened hospital stay [12]; however, several RCTs have reported no significant effect of TCZ against COVID-19 [25, 26]. This could be attributed to IL-6 pathway inhibition only being insufficient under critical conditions. Recently, Van den Eynde et al. reported that the hazard ratio of death was significantly lower in the combined therapy of steroid pulse and TCZ group than in the control group (< 1), which was lower than that in the steroid pulse only group and the TCZ only group [27]. Given the various side effects of long-term steroid administration, including susceptibility to infection, combining TCZ and steroid pulse therapy to quickly achieve strong immunosuppression, followed by a rapid decrease of the steroid dose, was considered better than long-term administration of moderate doses.

The combination of TCZ and steroid pulse therapy in our non-HD patients markedly decreased CRP and improved oxygen demand immediately after administration. Although CRP is inadequate indicator for treatment of COVID-19 because CRP production by IL-6 pathway is suppressed by TCZ, LDH, which indicates lung injury and severity of COVID-19 [28], also showed a decreasing trend. Serum LDH elevation not only reflect lung injury itself but also hyperinflammation status due to hyperactivation of macrophages and thrombotic microangiopathy following vascular endothelial damages [28]. This suggested that the early and potent suppression of the cytokine storm by combination of TCZ and steroid pulse therapy resulted in the prevent from inflammation, microangiopathy and lung fibrosis (Fig. 1). All deaths in the standard therapy group were associated with bacterial or fungal infections; contrastingly, there were no secondary infection cases in the TCZ + mPSL pulse group. This indicates we succeeded in reducing the infection risk by adding TCZ and tapering the steroid dose in the earlier treatment phase. All patients in both groups underwent intubation or HFNC; therefore, respiratory management did not affect the risk of infectious complications (Table 1).

Patients with kidney disease had worse COVID-19 outcomes [4]; specifically, HD patients showed a mortality rate of 14% [5]. Compared with healthy individuals, HD patients present with impaired immune function caused by immune disorders in both innate and adaptive immune systems, including altered monocyte phenotypes, reversal of the CD4 + /CD8 + ratio, and a significant decrease in naive T cells [7]. Additionally, the number of comorbidities in HD patients may affect their prognosis. In our study, all the patients who required an oxygen mask and respiratory management had non-diabetic and diabetic nephropathy, respectively. Furthermore, HD induces inflammation; specifically, IL-6 levels are increased after dialysis [29] Moreover, patients with diabetic nephropathy are particularly susceptible to inflammatory reactions induced by IL-6 [30], which suggests that IL-6 may be a key mediator in HD patients with COVID-19. Recently, Abe et al. reported that TCZ was effective in two cases of ARDS due to COVID-19 in HD patients with diabetic nephropathy [31]. Taken together, these findings suggest that the combination of TCZ with steroid pulse therapy would be more effective in HD patients with COVID-19. In our study, all three critical HD patients with COVID-19 who were treated with standard therapy died. Contrastingly, two critical HD patients who received TCZ and mPSL pulse therapy survived even though their oxygen demand exceeded 8 L/min. Two patients in the standard therapy developed secondary infections and died while one patient in the TCZ + mPSL pulse group developed secondary infections and survived (Table 3). Among the two patients with secondary infection in the standard therapy group, one underwent steroid treatment for a longer duration because of a lack of improvement in the oxygen demand due to organizing pneumonia. In the other case, only TCZ was added to dexamethasone. However, the oxygen demand did not decrease after TCZ administration, which resulted in organizing pneumonia with higher KL-6 levels. Subsequently, infection occurred due to prolonged steroid administration for uncontrolled organizing pneumonia. For all patients in the TCZ + mPSL pulse group, combination therapy of TCZ and steroid pulse could have suppressed excessive inflammation at an early stage, as well as prevented organizing pneumonia and lung fibrosis. Therefore, early steroid dose reduction may reduce the infection risk and improve the prognosis. Furthermore, the oxygen demand improved from 8 to 0–1 L/min within about 1 week, with a marked improvement in inflammatory response by combining TCZ and steroid pulse. This suggests that early and potent suppression of cytokine storm is effective in critical HD patients with COVID-19 (Figs. 2, 3).

This study has several limitations. First, we used the oxygen demand as a pulmonary oxygenation index, which is less objective than the P/F ratio (we could not calculate the P/F ratio given the difficulty of frequent arterial gas measurements). Further, this was a retrospective study with a small sample size; moreover, we did not compare between steroid pulse alone and the combination of TCZ and steroid pulse. There is a need for future RCTs comparing steroid pulse alone, TCZ alone, and the combination of TCZ and steroid pulse.

## Conclusion

In conclusion, compared with standard therapy, the combination of TCZ with steroid pulse therapy improved the survival rate without significant adverse events in critical HD and non-HD patients with COVID-19 through strong suppression of systemic hyperinflammation in the early phase and early steroid dose reduction, which reduced the infection risk and improved the prognosis.

## Data Availability

The datasets generated during and/or analyzed during the current study are available from the corresponding author on reasonable request.
